# In-situ electronics and communications for intelligent energy storage

**DOI:** 10.1016/j.ohx.2022.e00294

**Published:** 2022-03-12

**Authors:** Joe Fleming, Tazdin Amietszajew, Alexander Roberts

**Affiliations:** Coventry University, United Kingdom

**Keywords:** Cell instrumentation, Smart cells, Electronics, Thermistors, Cell safety, Cell performance, Cell formation, Thermal monitoring

## Abstract

Lithium-ion batteries are increasingly common in high-power, safety–critical applications such as aerospace, spaceflight, automotive and grid storage. The voltage and power specifications of such applications usually require large numbers of individual cells combined in series and parallel to form a battery pack. It is then the role of the Battery Management System (BMS) to monitor these cells condition and ensure they remain within safe operating limits. To minimise cost and complexity, it is typical to monitor only a fraction of the cells in a battery pack. This creates potential safety and reliability issues and requires conservative limits imposed on the overall system to ensure safe operation. This is insufficient in high-power, safety–critical applications and thus alternative approaches to battery management are required. Here we demonstrate the development of novel miniature electronic devices for incorporation in-situ at a cell-level during manufacture. This approach enables local cell-to-cell and cell-to-BMS data communication of sensor data without the need for additional wiring infostructure within a battery module assembly. The electronics firmware and hardware integration within the cell’s electrode stack is demonstrated to function after triggering post cell formation and through cycling and electrochemical impedance analysis. This work shows that the proposed approach has a negligible impact on the cells’ performance and highlights a new technique for active monitoring of the cell’s in-situ conditions. This research will enable new methods of cells characterization and monitoring for optimum electrochemical and thermal performance while improving system safety.

## Graphical abstract

**Figure:**
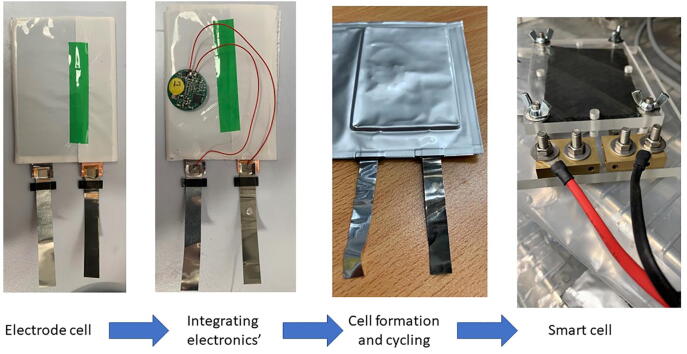

## Specifications table

Please replace the italicized instructions in the right column of the table with the relevant information about your hardware.

**Table:** 

Hardware name	*In-situ* electronics and communication for intelligent energy storage
Subject area	• Engineering and Material Science Chemistry and Biochemistry General
Hardware type	• Measuring physical properties and in-lab sensors Field measurements and sensors
Closest commercial analog	• Measuring physical properties and in-lab sensors Field measurements and sensors
Open-source license	Creative Commons - Attribution - ShareAlike 3.0
Cost of hardware	*£25*
Source file repository	https://doi.org/10.17632/z6nmc3kcy8.3

## Hardware in context and description

Lithium-ion cells are often the first choice of technology for large scale energy storage, electric vehicles, and portable electronics. Depending upon the chemistry selected and application requirements, such benefits include a high energy density, no memory effect and high nominal cell voltage. But strict electrochemical and thermal management strategies must be implemented for safety, reliability, and performance, as the electrochemical systems are extremely sensitive and complex systems. Such performance indicators include, temperature and cell voltage, but these parameters are typically not known in large scale battery systems at a cell level, due to the complexity and mechanical limitations of adding a wiring harness to a module. Consequently, an unmanaged system can lead to a catastrophic system failure and performance reduction [1]. Therefore, significant effort is undertaken to mitigate the effects of performance degradation and to understand key environmental conditions such as temperature, voltage, current and gassing within a cell that can give an indicator of a cells current state-of-health (SoH).

The need for accurate information regarding the state of health of cells during run-time operation has had several publications regarding the integration of various sensing devices including, resistance temperature detectors (RTD’s) [2], thermocouples [3] thermistor arrays [4], optical sensors [5] and reference electrodes [6], [7]. However, these solutions often egress from the cells’ enclosure, which would not be suitable for a mainstream application. For example - a Tesla model 3 pack contains approximately 2976 cells a Nissan leaf 24 kWh has 192 pouch cells. Consequently, such a high number of cells would require a significant increase of sensors cabling for monitoring of each cell. Adding significant cost, weight, and infrastructure complexity. Therefore, the 2019 Nissan Leaf’s battery pack can only rapid charge once in a 24-hour period[8], due the limited knowledge of the core temperatures of each cell within the battery module and a lack of long-term understanding of the effects of such a performance increase has on the system.

This study aims to implement powerline communication (PLC), at a cell level, with the intention to fully integrate the circuit into the cell during manufacturing. Our unique approach utilising PLC offers a viable alternative to signal transmission in harsh and space restricted systems due to the reduced requirement of added sensing or communication wires. The technology is often used and adopted for the use in home electrical networks for distributed broadband, satellites sensor communication for weight reduction and photovoltaic systems for remote data transmission. PLC operates by injecting a carrier signal onto a power line, typically in AC and/or DC networks, the carrier signal is then modulated with data, thus enabling communications.

Several researchers have attempted various methods of integrating communication at a cell level; including capacitive coupling [9], [10], wireless radio [11] and to some degree low frequency power-line communication [12], [13], [14] but none of these solutions develop powerline communication in-situ of a cell, previous work has mounted externally and therefore sensor readings may not reflect the true state of health of the cell and the impact of embedded such technology within a pouch cell is undefined. There are a number of challenges associated with introducing sensors into Li-ion cell systems, including a harsh chemical environment, thermal effects causing expansion of materials and temperature gradients and electrical and mechanical interference can be encountered during normal cell usage.

These voltages are high and of a lower frequency and may cause damage in over-potential and therefore, an absolute minimum would be to have the technology developed compared with a typical radio frequency receiver will enable a signal level within the microvolt level (−81 dBm).

Our proposed solution is to utilise the anode and cathode connection within the cell for transmission of data, in essence connecting our device across the battery terminals in-situ of the cell. The benefits of powerline communication are that the exiting power bus bars are used as the transmission medium, thus significantly reducing the complexity of implementing a system of intelligent cells in a battery module. Furthermore, such modifications to a cell could have a detrimental effect on cell performance, our study shows through time and frequency domain analysis that such effects are negligible. Consequently, this work on cell instrumentation methodology has the potential to facilitate significant advances in battery technology in which active electronics and sensors are embedded during manufacturing. This work will enable an intelligent smart cell which can communicate with the battery management system over a pre-existing connection infrastructure.

## Design files summary

**Table:** 

Design file name	File type	Open-source license	Location of the file
*PCB Gerber*	CAD	CC BY NC 3.0	https://doi.org/10.17632/z6nmc3kcy8.3
PCB Schematic	Drawing	CC BY NC 3.0	
PCB Layout	Drawing	CC BY NC 3.0	
PCB Firmware	C Firmware	CC BY NC 3.0	
PCB BOM	File	CC BY NC 3.0	

## Bill of materials summary

**Table:** 

No	Qty	Description	Reference	Supplier	Part Number	Unit Cost
1	1	PIC16F1825	IC2	Microchip	PIC16F1825-I/ML	£ 1.50
2	1	UART to DC SIG60	IC1	Yamar	SIG60	£ 5.00
3	1	Dp-Charge Pump/General Purpose 3.3 V	IC3	Linear Technology	LTC3240EDC-3.3#TRMPBF	£ 0.75
4	1	Generic Thick Film Surface Mount Fixed Resistor, 0402, 1.2 K	R3	Eurocircuits	GPR04021K2	£ 0.01
5	2	GenericThick Film Surface Mount Fixed Resistor, 0402 18R	R1,5	Eurocircuits	GPR040218R	£ 0.01
6	4	Generic Thick Film Surface Mount Fixed Resistor, 0402 100 K	R7,10,11,12	Eurocircuits	GPR0402100K	£ 0.01
7	1	Generic Thick Film Surface Mount Fixed Resistor, 0402 470R	R2	Eurocircuits	GPR0402470R	£ 0.01
8	1	Generic Thick Film Surface Mount Fixed Resistor, 0402 10R	R6	Eurocircuits	GPR040210R	£ 0.01
9	3	Generic Capacitor, 0402, ceramic, 1nF	C1, 16,18	Eurocircuits	GPC0402102	£ 0.01
10	1	Generic Capacitor, 0402, ceramic, 220pF	C3	Eurocircuits	GPC0402221	£ 0.01
11	1	Generic Capacitor, 0402, ceramic, 2.2nF	C2	Eurocircuits	GPC0402222	£ 0.01
12	2	Generic Capacitor, 0603, ceramic, 1uF	C8,9	Eurocircuits	GPC0603105	£ 0.01
13	1	Generic Capacitor, 0402, ceramic, 1uF	C17	Eurocircuits	GPC0402105-16	£ 0.01
14	1	Generic Capacitor, 0402, ceramic, 4.7uF	C10	TDK	C1005X6S0G475M050BC	£ 0.01
15	2	Generic Capacitor, 0402, ceramic, 100nF	C4,11	Eurocircuits	GPC0402104	£ 0.01
16	1	Inductor, Ferrite, 22uH, 0603	L1	Taiyo Yuden	LBMF1608T220K	£ 0.01
17	1	4 MHz Oscillator CMOS SMD	Q3	EPSON	X1G004171000912	£ 0.41
18	1	Inductor, 0603, High-Q, 8.2 uH	L2	TDK	MLF1608E8R2K	£ 0.20
19	1	Inductor, 0603, High-Q, 4.7 uH	L4	TDK	MLZ1608M4R7WT	£ 0.20
20	1	Inductor, 0603, High-Q, 15 uH	L3	Coilcraft	0603LS-153XJLB	£ 0.20
21	1	Generic Capacitor, 0603, ceramic, 68 pF	C12	Eurocircuits	GPC0603680	£ 0.20
22	2	Generic Capacitor, 0603, ceramic, 47 pF	C13,14	Eurocircuits	GPC0603470	£ 0.20
23	1	Generic Capacitor, 0603, ceramic, 680 pF	C15	Eurocircuits	GPC0603681	£ 0.20
24	1	Generic Capacitor, 0603, ceramic, 330 pF	C19	Eurocircuits	GPC0603331	£ 0.20
25	1	Thermistor 0402 100kΩ	TH1	Vishay	NTCS0402E3104JHT	£ 0.05
26	4	Small Signal Schottky Diode, Single, 30 V, 70 mA, 350 mV, 500 Ma	D4,5,6,7	ON SEMICONDUCTOR	NSR0140P2T5G	£ 0.05

## Build instructions

**Fig. 1:**
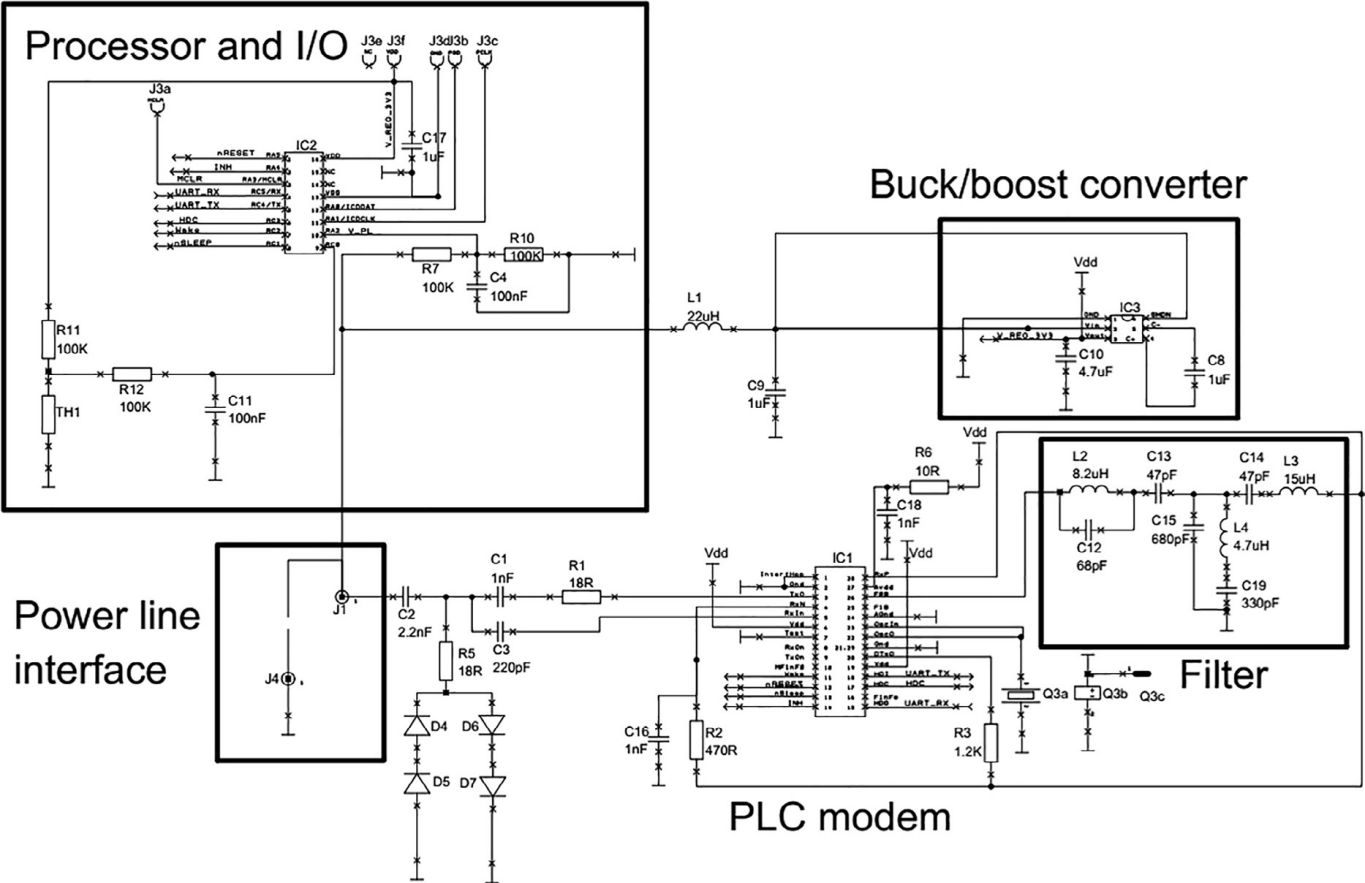
Illustration of the complete Electronics power line communication circuit for in-situ monitoring of energy storage.

To demonstrate our approach for injecting and extracting data through the cell an application specific integrated circuit (ASIC) SIG60 (Yamar©) was used as the power line communication transceiver technology. Additionally, a microcontroller (PIC18LF25K50) was used to monitor *in-situ* temperature and cell potential. A thermistor is used to monitor the temperature, this has the advantage of a high temperature sensitivity, consequently meaning the analogue instrumentation is minimal compared with a thermocouple or resistance temperature detector (RTD). A complete circuit diagram is illustrated in Fig. 1.

Lastly, the integrated circuits used in this design require a specific voltage range to maintain correct functionally and remain within the cells manufacture’s operation specification. Therefore, a charge pump conditions the battery voltage for compatibility with the integrated circuits, enabling a full operating range of 1.8 – 6 Vdc. A PCB layout of the circuit and bill of materials (BOM) are contained within the supplemental section.

This circuit configuration will provide a relative temperature accuracy of around 0.1 °C and a voltage accuracy of 20 mV, these values can be further increased with correct calibration with a reference source and adding a stable voltage reference alongside the circuitry.

### In-situ electronics protection

The circuit assembly was conformally coated with a polymer protective coating of Parylene-C (5 µm) using a PDS 2010 Labcoter® 2 (Specialty Coating Systems). The result was a uniform non-porous layer, protecting the sensor from electrical, chemical and mechanical interactions This has been shown to be effective as a barrier protectant within the electrochemical system in previous work conducted within this group [4], [5], [6], [7], [17]. To test the integration feasibility within a pouch cell, the connections to power the circuit were soldered to the anode and cathode tabs and a strain relief Kapton tape was placed over the wires. This method connects the electronics in parallel with the battery system. Full details of the electronics, printed circuit board and bill of materials is given within supplemental information.

### Firmware

For demonstration purposes a Microchip© 8-bit microcontroller was used as the host for the battery management firmware, the code was written in ANSI C language and developed within MPLAB studio environment. The firmware is simple with three main functions: a power state-machine, sensor measurement and communications. A watchdog timer is used as time triggered clock; therefore, the firmware will reset if a software or hardware hang-up fault occurs during run time operation, the firmware can then place the unit in a safe state if the fault is not resolved during the reset operation. This WDT an extremely useful feature for safety critical applications when used in this way as the unit is not accessible to reset.

### Software

A windows application written in C# was developed to log and interpret the signals transmitted through the powerline communication network. Using a standard terminal software is possible however the data would need to be parsed before interpretation could take place. This is because ASCII transmission consumes an increased amount of power during data exchanges due to the increased number of bytes required. The complete software code is available within the supplementary information section.

### Cell cycling and characterisation

**Fig. 2:**
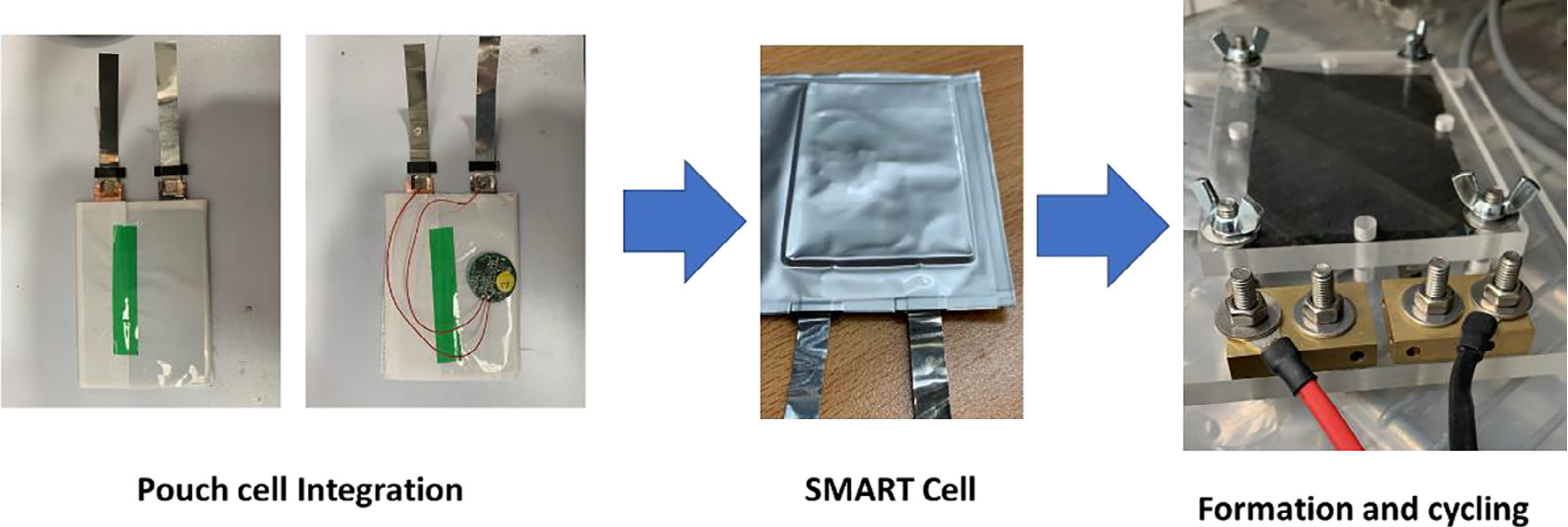
Block diagram Illustration and experimental setup of the power line communication system for an automotive module.

To evaluate the universal approach of embedding *in-situ*electronics as shown within Fig. 2 we have selected to demonstrate this technology within lithium-ion pouch cells, while pouch formations are more flexible in form factor, enabling varied applications, they require significant thermal and mechanical support from the battery module structure.

The pouch cells evaluated for the in-situ sensors application were 21-layer A7 sized with a nominal capacity of 1.4Ah, consisting of a lithium nickel manganese cobalt (NMC-622) cathode, graphite anode and 8.6 mL 1 M LiPF_6_ electrolyte solution. A batch of cells was manufactured in-house using Coventry University cell prototyping line. To mimic the typical cell behaviour encountered during its operation we have performed test cycles consisting of constant-current (CC) followed by constant-voltage (CV) charge and constant-current (CC) discharge, which is a standard for the lithium-ion chemistry. Furthermore, Electrochemical Impedance Spectroscopy (EIS) was applied to the device to understand performance degradation which may not become apparent in CC/CV cyclic aging analysis.

All experiments were conducted in an environmental chamber (Binder®) maintaining an ambient temperature of 25 °C. The 1.4Ah pouch cells were cycled at C/3 and down to 2.8 V and the formation protocol used for the instrumented cells uses the following protocol; 25 °C, C/20 to 4.2 V, CV to C/100 or 4 h, CC C/20 to 2.5 V, two cycles. The cell cyclers used for this experimentation were VMP3 multi-channel potentiostats (*Bio-Logic Science Instruments®*). Calibration of all sensing equipment was conducted where possible near the time of the experiment.

## Validation and characterization

We present our results in three parts. We first show the integration of the sensing technology within a pouch cell. Then we show and discuss the electrochemical impact and demonstrate the performance through time and frequency domain analysis.

### In-situ integration and formation

**Fig. 3:**
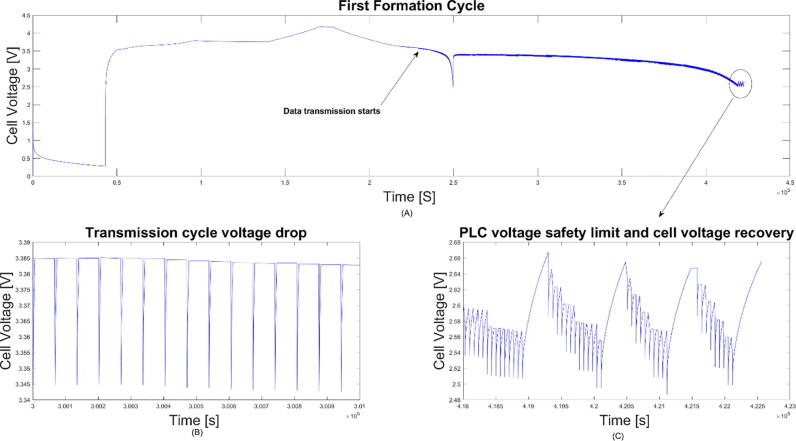
Illustration of a cell voltage profile during formation and the following 50 h rest.

The cell manufacturing process and the quality of this process plays a critical part in the future performance and reliably of cells. One particularly important step is the cell formation process. After filling with electrolyte, the cells are put under a formation procedure – during this critical phase, the solid electrolyte interface layer is formed through charging and discharging the cell at a controlled rate. The cell formation can take days depending on chemistry and is carefully controlled. Initial experimentation showed that transmitting data through the system would cause the formation process to fail due to the extra current drawn by the parallel load of the transmission circuit. This was overcome by enabling a time-delay within the system on first power-up. In essence, the device will not transmit data until after the formation process, not to affect the formation process. Fig. 3 shows the formation of a lithium-ion cell with the power-line communication technology implemented. It can be seen the extreme low-power mode is not visible at the cell voltage level during formation. Additionally, a low-voltage limit off switch has been implemented, to avoid a situation where over-discharge may cause damage by pushing the cell past a nominal discharge level. This switch has been set to have a trigger threshold of 2.6 V which can be adjusted in the firmware to suit a particular cell chemistry, therefore the device will only transmit data if the cell voltage measured is above the lower-cut of frequency.

### Time domain analysis - standard cycling

Here we evaluate the stability of the sensor for long-term, operando measurement in the harsh environment of the cells. We also show that the system integration of the distributed sensor does not affect the performance of the cells. In particular, we evaluate the sensors readings stability and the cell capacity retention, a commonly agreed indicator of the Li-ion cells’ SoH. Typically, cells are considered at their end-of-life when the capacity falls below 80 % of the rated value.

**Fig. 4:**
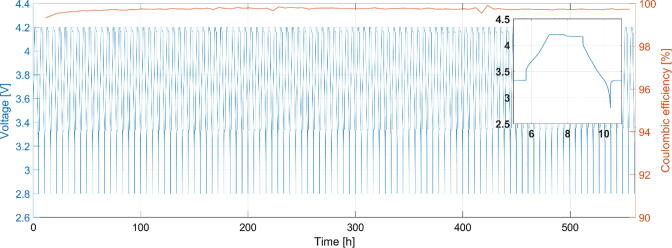
Potential and efficiency readings over 100 representative full cell cycles (over 24 days), under continuous data transmission, obtained using a precise (100 μVolt resolution) electrochemical channel. Cells were cycled using their standard cycling profiles between 2.8 V and 4.2 V. The insert shows focused single charge/discharge cycle. It is visible that the system retains good stability and high efficiency.

In the first instance data was sent at a continues rate through the power line transmission circuit, thus emulating a network with multiple communication nodes present. The cells where utilised within the safety limits of operation specified by the cell chemistry. The cells were monitored over 100 cycles under continuous data transfer. They behaved as expected when cycling, retaining their base capability of energy storage and power delivery. The average Coulombic efficiency of the system over 100 cycles was at 99.75%, including the energy losses for continuous data transmission. The resulting CC/CV curves, shown in Fig. 4 below, confirm stable charge/discharge behavior of the system.

### Electrochemical and power line frequency domain analysis

**Fig. 5:**
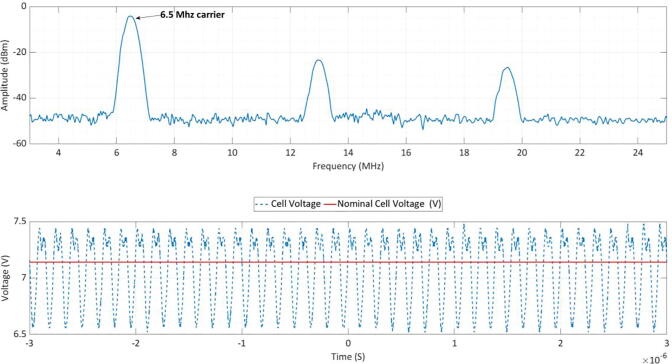
Spectral power distribution on the DC power bus while the PLC circuit is powered by the batteries in a 2S configuration.

A frequency spectrum analyzer was used to measure the fundamental harmonic on the cell as illustrated within Fig. 5 during a transmission cycle. The PLC circuit has a center frequency of 6.5 MHz as expected but the additional harmonics may cause interface with other electrochemical frequency analysis methods that could be use in parallel with PLC technology such as electrochemical impedance response and electrochemical harmonic response (EHA). The PLC unit applies of 600 mV 6.5 MHz carrier frequency, this extremely high frequency and short duration pulse has shown to have limited impact on the cell performance as shown within our work, but safety limits are in place that can stop a transmission if over-voltage is expected.

The absolute minimum voltage signal that our technology can receive is 20 mV but it can drive a signal up to 600 mV, Other researchers use carrier amplitudes including 350 mV [15] and 200 mV [16] but the carrier frequencies are within the Khz frequency band, while ours is a much higher frequency band (6.5 MHz+), meaning the on-time during possible over-potential events is lower and higher data bandwidth can be achieved. It would be beneficial under most circumstance to reduce the amplitude to an absolute minimum to reduce any over or under potential damage to cells, most radio frequency receivers will work down to the micro-volt range and such future work should look at reducing signal amplitude in large battery networks.

The electrochemical impedance response is another important parameter indicative of the cells’ internal health. The cells with the integrated *in-situ* electronics system were analysed through Electrochemical Impedance Spectroscopy [18], a highly sensitive measurement method used to observe the impedance response of a system over a range of alternating current (AC) signal frequencies, allowing for energy storage and dissipation properties comparison. It must be noted that EIS was conducted upon these cells and the data was not aligned with a standard cell during a continues data transmission. This is due to the high current burst effects of the electronics, further investigation is required if on-line EIS measurements are required, this could be achieved by disconnecting the electronics or by not transmitting data during EIS measurement during this period.

**Fig. 6:**
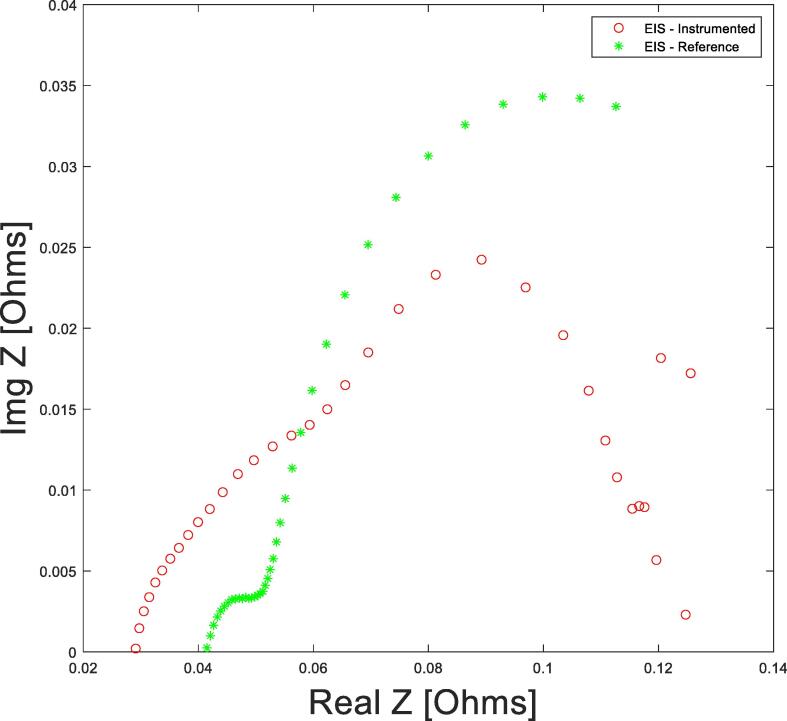
Illustration of the Electrochemical impedance response of an instrumented and standard lithium-ion.

We have shown it is possible to conduct EIS while the unit is not transmitting data and we also show that the parallel circuit has a negligible effect on the overall system performance as illustrated within Fig. 6. The EIS comparisons do look different but are representative of what a typical response curve expected of a cell. The low frequency zone of the instrumented EIS plot, indicative of the diffusion region displays noise, this can be explained due to the parrel loading of the electronics system, consequently adding a further complex resistance. Also, the unit in sleep mode still draws power and thus maintaining a constant state of charge is difficult.

**Fig. 7:**
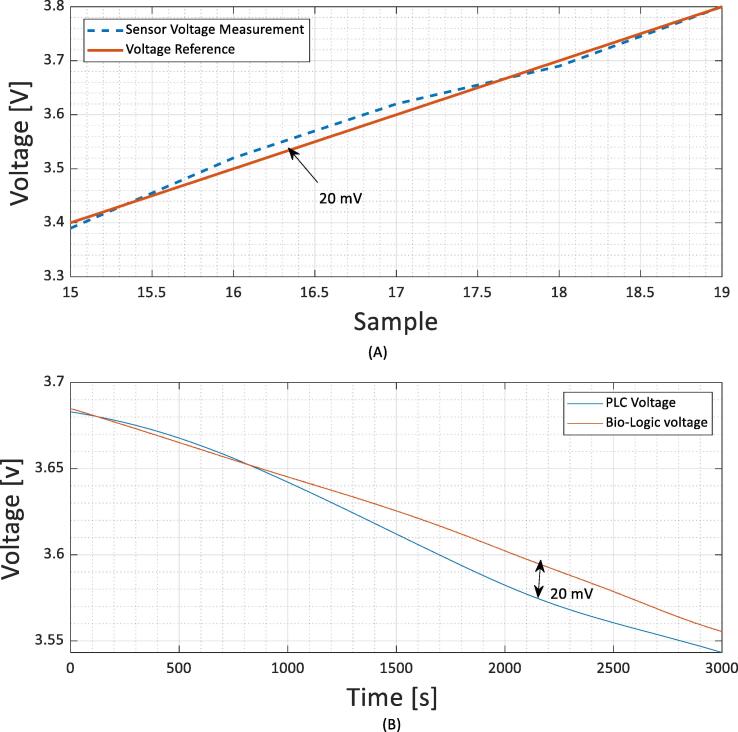
Illustration of the sensor in-situ of a cell (a) and (b) Illustration of the sensor compared versus a calibrated voltage source.

Lastly Fig. 7(A) illustrates the voltage measured by the power-line communication board while discharging a cell using a bio-logic unit and the voltage measured using the bio-logic unit. The raw voltage measurement is averaged 8 times using successive measurement modes. The ADC reference used is an internal-band gap diode and a voltage divider is used to reduce the voltage input. The error calculated from the datasheet of the microcontroller is estimated to be ±26 mV (Integral error + Differential error + offset error and gain error) with a 10-bit resolution and a 4 V reference. The voltage measured by the unit show a clear 20 mV difference in measurement accuracy which can be improved by adding a high precision voltage reference or increase the resolution of the ADC. We have also measured the stability of the voltage measurement using a calibrated voltage source as illustrated within Fig. 7(B), the data is in clear alignment with the reference and within the specification given with the datasheet and is more than acceptable for a battery management system to use as a monitoring device.

## Conclusions

The objective of this study was to develop and enable *in-situ* communication and measurement system for lithium-ion cells and characterise the effect upon the electrochemical performance. We propose a widely applicable smart cell concept enabling unprecedented in-situ and operando monitoring of cells. Furthermore, this study has also shown that injecting a signal through a battery system via the powerline is possible and has limited if no effect upon the electrochemical performance. Moreover, this study has physically integrated the electronics with a full pouch cell assembly, detailing additional steps to the production process. To thoroughly evaluate the smart cells design, we used several characterization methods Electrochemical impedance spectroscopy and repetitive cycling over a sustained period was deployed to assess the cell’s electrochemical performance as on-par with unmodified cells.

Our future work involves the integration of such devices within large scale energy storage systems, such as those used with automotive EV modules. However, challenges and unknowns still exist which include the harsh electromagnetic noise from the drive train and surrounding environment, to date much work has been carried out within labs environments or using module cyclers, which do not reflect the true state of use. Moreover, cells are configured into packs in serial and parallel configurations, bus bars are used to connect the cells together with welded joints. However, the cells, bus bars, weld joints and surrounding electronics may cause signal reflections and induce noise which will affect signal integrity and reliability of a communication system. A trade of between useful data and power consumption needs to be taken into account when designing future battery management algorithms, the device will have a power draw on each cell, so cell balancing will also be an important factor. Moreover, this could increase the circuitry and thus require the need for an application specific circuit to be developed, which would be a high capital expenditure so this initial research is important in validating that such an investment would be beneficial to the energy storage market.

This work shows that our proposed in-situ sensing approach has the potential to drive improvement in both performance and operational safety mapping. The thermal and voltage data that can be gathered with the use of the smart cells represents a vital source of key information, critical for the battery management systems to maintain a most optimal performance and an up-to-date understanding of the cells State of Health during deployment in real-life scenarios. Finally, it is foreseen that the electronic capabilities implementation methodology developed here will support the design, research and rapid prototyping of new cells and smart battery modules, enabling considerably greater performance to be safely harnessed from these increasingly prevalent Li-ion energy storage systems.

### CRediT authorship contribution statement

**Joe Fleming:** Writing – review & editing. **Tazdin Amietszajew:** . **Alexander Roberts:** .

## Declaration of Competing Interest

The authors declare that they have no known competing financial interests or personal relationships that could have appeared to influence the work reported in this paper.
